# Differential engagement of the posterior cingulate cortex during cognitive restructuring of negative self- and social beliefs

**DOI:** 10.1093/scan/nsad024

**Published:** 2023-05-02

**Authors:** James Agathos, Trevor Steward, Christopher G Davey, Kim L Felmingham, Sevil Ince, Bradford A Moffat, Rebecca K Glarin, Ben J Harrison

**Affiliations:** Melbourne Neuropsychiatry Centre, Department of Psychiatry, The University of Melbourne, Carlton, Victoria 3053 Australia; Melbourne Neuropsychiatry Centre, Department of Psychiatry, The University of Melbourne, Carlton, Victoria 3053 Australia; Melbourne School of Psychological Sciences, The University of Melbourne, Parkville, Victoria 3010, Australia; Department of Psychiatry, The University of Melbourne, Parkville, Victoria 3010, Australia; Melbourne School of Psychological Sciences, The University of Melbourne, Parkville, Victoria 3010, Australia; Melbourne Neuropsychiatry Centre, Department of Psychiatry, The University of Melbourne, Carlton, Victoria 3053 Australia; Melbourne School of Psychological Sciences, The University of Melbourne, Parkville, Victoria 3010, Australia; Melbourne Brain Centre Imaging Unit, Department of Radiology, The University of Melbourne, Parkville, Victoria 3010, Australia; Melbourne Brain Centre Imaging Unit, Department of Radiology, The University of Melbourne, Parkville, Victoria 3010, Australia; Melbourne Neuropsychiatry Centre, Department of Psychiatry, The University of Melbourne, Carlton, Victoria 3053 Australia

**Keywords:** cognitive restructuring, posterior cingulate cortex, default mode network, negative self-beliefs, social cognition

## Abstract

Negative self-beliefs are a core feature of psychopathology, encompassing both negative appraisals about oneself directly (i.e. self-judgment) and negative inferences of how the self is appraised by others (i.e. social judgment). Challenging maladaptive self-beliefs via cognitive restructuring is a core treatment mechanism of gold-standard psychotherapies. However, the neural mechanisms underlying the restructuring of these two kinds of negative self-beliefs are poorly understood. Eighty-six healthy participants cognitively restructured self-judgment and social-judgment negative self-belief statements during 7 Tesla functional magnetic resonance imaging scanning. Cognitive restructuring broadly elicited activation in the core default mode network (DMN), salience and frontoparietal control regions. Restructuring self-judgment relative to social-judgment beliefs was associated with comparatively higher activation in the ventral posterior cingulate cortex (PCC)/retrosplenial cortex, while challenging social-judgment statements was associated with higher activation in the dorsal PCC/precuneus. While both regions showed increased functional connectivity with the supplementary and pre-supplementary motor areas during restructuring, the dorsal PCC displayed greater task-dependent connectivity with distributed regions involved in salience, attention and social cognition. Our findings indicate distinct patterns of PCC engagement contingent upon self- and social domains, highlighting a specialized role of the dorsal PCC in supporting neural interactions between the DMN and frontoparietal/salience networks during cognitive restructuring.

## Introduction

Cognitive-behavioral models of psychopathology posit that biased, maladaptive beliefs about the self, e.g. that one is incompetent, or not worthy of being loved (Disner *et al.*, 2011), are a core underlying component of mood and anxiety disorder symptomatology (Beck and Clark, 1988). The malleability of self-beliefs, or the extent to which individuals believe that they can be changed, has been noted as a predictor of depressive symptom severity (Everaert, 2021), as well as treatment response to psychotherapy (de Castella *et al.*, 2015). Consequently, ‘cognitive restructuring’ techniques, which aim to identify and challenge maladaptive cognitions and self-evaluations by considering the factual experience and personal experiences that counter them (i.e. through therapist-guided Socratic questioning; Leahy and Rego, 2012; Clark and Egan, 2015), are a key treatment mechanism of gold-standard psychotherapies, such as cognitive-behavioral therapy (CBT; Clark and Egan, 2015). Indeed, reducing maladaptive self-beliefs during CBT for social anxiety disorder is a predecessor to subsequent reductions in social anxiety severity (Goldin *et al.*, 2013; Gregory *et al.*, 2018).

Functional brain imaging research over the past two decades has significantly advanced our understanding of how self-related mental processes are supported by the activity of large-scale brain systems. Studies have consistently implicated the ‘default mode network’ (DMN) in self-related processes: a higher-order neural system that has a broad orchestrating role in human cognition and behavior by virtue of its dense and unique brain-wide connectivity (Raichle *et al.*, 2001; Raichle and Snyder, 2007; Buckner *et al.*, 2008; Margulies *et al.*, 2016; Alves *et al.*, 2019). The DMN’s contributions to self-related processes arise from its dynamic integrative processing of the internal (self) and external environments (Davey *et al.*, 2016; Putica *et al.*, 2021; Davey and Harrison, 2022; Dixon *et al.*, 2022). In this respect, the medial prefrontal cortex (mPFC) and posterior cingulate cortex (PCC) have been identified as central hubs of the DMN, with the ventral PCC integrating multiple inputs from temporoparietal cortical areas to coordinate mental representations of the self and others (Carhart-Harris *et al.*, 2014), while the mPFC modulates this activity by directing selective attention and gating these representations into conscious awareness (Andrews-Hanna *et al.*, 2010; Davey *et al.*, 2016).

It has been suggested that the DMN may support the challenging of negative self-beliefs by acting in concert with frontoparietal control regions, with the DMN holding and directing attention to mental representations of the self and frontoparietal regions supporting the modification of these self-representations (Goldin *et al.*, 2009; Dixon *et al.*, 2020). However, it is not yet clear whether this relationship is generalizable across different kinds of self-beliefs. There is evidence for distinct patterns in DMN engagement between direct self-appraisal (i.e. thinking about oneself) and reflected self-appraisal (i.e. thinking about how the self is perceived by others, e.g. ‘Other people find me boring’)—while both are supported by the core self-network comprising the mPFC, ventral PCC and inferior parietal lobule (IPL), reflected self-appraisal is characterized by comparatively greater activation and connectivity of the ventral PCC and left IPL (Delahoy *et al.*, 2022). This finding may reflect that distinct higher-order processes are required for perspective shifting and mentalizing during reflected self-appraisal (Delahoy *et al.*, 2022). As such, we might expect that both forms of self-appraisal draw upon similar cognitive processes, such as evaluating one’s own characteristics and drawing upon self-relevant information from episodic memory (Conway, 2005)—however, reflected self-appraisal may be distinguished by mentalizing processes that allow individuals to consider how they are perceived by others.

In addition to the ventral PCC, previous studies have highlighted that the dorsal PCC may also support mentalizing about the actions and thoughts of others, including inference of others’ mental states (Spunt *et al.*, 2011; Bellucci *et al.*, 2020). As such, while it appears that self-appraisal is broadly characterized by robust PCC engagement, the specific contributions of PCC subregions remain to be clarified. The extent to which this circuitry may also support the challenging of social-judgment (i.e. involving reflected self-appraisal) self-beliefs relative to self-judgment (i.e. direct self-appraisal) is equally not well understood. Furthermore, past brain network parcellation and functional connectivity studies note that the ventral and dorsal PCC subcomponents are associated with different subsystems within and beyond the DMN (Andrews-Hanna *et al.*, 2010, Yeo *et al.*, 2011; Andrews-Hanna et al., 2014), and whether these might also differ during cognitive restructuring is yet to be explored.

The aim of the current study was to characterize the neural basis of cognitive restructuring of self-judgment *vs* social-judgment beliefs. It was hypothesized that cognitive restructuring of negative self-beliefs broadly implicates core DMN and frontoparietal control regions. We further hypothesized that the ventral PCC may play a greater role during the challenging of social-judgment self-beliefs (requiring reflected self-appraisal) relative to self-judgment beliefs (requiring direct self-appraisal). We tested this prediction by directly comparing self- *vs* social judgments in terms of their evoked activity during cognitive restructuring and by investigating potential regional differences in terms of their functional interactions with the broader restructuring network.

## Materials and methods

### Participants

Eighty-six healthy adults were recruited for this study. A subset of these participants (*n* = 42) was included in a previous report (Steward *et al.*, 2022). All participants met the following eligibility criteria: (i) aged between 18 and 40 years, (ii) did not meet criteria for any psychiatric disorders as screened using the Mini-International Neuropsychiatric Interview (Sheehan *et al.*, 1998) and (iii) had no magnetic resonance imaging (MRI) contraindications (e.g. pregnancy, metallic implants or claustrophobia). All participants were competent English speakers and had normal or corrected-to-normal vision. Participants provided written informed consent and attended a single testing session at the Melbourne Brain Centre Imaging Unit (MBCIU, The University of Melbourne, Parkville). This study was approved by the University of Melbourne Human Research Ethics Committee.

Eight participants were excluded from this initial sample due to poor physiological (cardiac and respiratory) data-recording quality (*n* = 3), excessive head motion (*n* = 4) and not having any trials in a given condition to facilitate comparisons between self- and social-judgment challenging *vs* repeating (*n* = 1). As a result, 78 healthy participants (35 females, 41 males, 1 non-binary and 1 undisclosed; age 24.24 ± 4.92 years) were included in the final functional MRI (fMRI) analyses.

### Experimental design

As detailed by Steward *et al.* (2022), participants were trained prior to scanning on cognitive restructuring skills using Socratic questioning techniques, such as active rebuttal, reinterpretation and perspective shifting (Beck and Dozois, 2011). After being shown a negative self-belief statement, participants were instructed to recall previous instances when the statement was not true or to argue against the statement using factual information. To confirm participants’ ability to carry out cognitive restructuring strategies, participants were asked to verbalize how they intended to mentally reframe example negative statements before undergoing scanning.

**Fig. 1.**
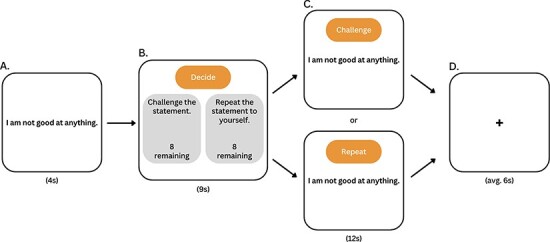
Cognitive restructuring paradigm. In each block, (**A**) a negative belief statement was presented for 4 s; (**B**) next, participants had 9 s to decide whether to challenge (CHAL) the statement using previously trained Socratic questioning techniques or to repeat (REP) the statement to themselves. During each block, participants were shown how many remaining choices they had for each option (CHAL or REP); (**C**) after their choice, participants either cognitively reframed or repeated the statement for 12 s; (**D**) a fixation cross was displayed for an average of 6 s before the next block began.

The cognitive restructuring task consisted of a single run containing 16 blocks (Figure 1). In each block, participants were first presented with a negative self-belief statement for 4 s. These comprised eight self-judgment and eight social-judgment belief statements. Next, participants were given 9 s to decide whether to challenge (i.e. cognitively reframe) or repeat the statement. To ensure an equal number of blocks for each condition, participants were instructed to only cognitively restructure half the statements and to repeat the remaining half of the statements. The remaining number of times that participants could challenge or repeat a statement was displayed alongside these two choices. Participants indicated their choices using an MRI-compatible control pad. After their choice, the same statement was displayed for 12 s, during which time participants engaged in their previously selected technique. If the participant chose to restructure the negative belief, a prompt reading ‘Challenge’ was displayed alongside the statement, and participants were instructed to mentally refute or reinterpret the negative statement throughout the entire 12 s (herein referred to as ‘CHAL’). If the choice were to repeat, a prompt reading ‘Repeat’ was shown with the negative statement, and participants were instructed to mentally recite the negative belief until the 12 s had expired (herein referred to as ‘REP’). Between each statement block, a fixation cross was presented for an average of 6 s to reduce carryover effects.

Prior to and following scanning, participants completed a 7-point self-rated questionnaire including the 16 negative statements presented during the cognitive restructuring task. Participants were instructed to indicate their level of agreement (1 = strongly disagree and 7 = strongly agree) with each statement. These statements were adapted from common negative self-beliefs described in the CBT literature (Beck and Dozois, 2011; Osmo *et al.*, 2018), including statements such as ‘I sometimes feel incompetent in the things I do’ and ‘I think that I’m a failure’. To verify the effectiveness of cognitive restructuring during the task, participant endorsement levels of negative self-beliefs pre- and post-scanning were compared using paired-sample two-tailed *t*-tests. Intra-task affective responses to the statements were not explicitly measured.

### Ultra-high-field image acquisition

Imaging was performed on a 7 Tesla (7T) research scanner (Siemens Healthcare, Erlangen, Germany) equipped with a 32-channel head coil (Nova Medical Inc., Wilmington, MA, USA). The functional sequence consisted of a multiband- (six times) and grappa (two times)-accelerated gradient-echo planar imaging sequence in the steady state [Repetition Time, 800 ms; Echo Time, 22.2 ms; pulse/flip angle, 45°; field of view, 20.8 cm; slice thickness (no gap), 1.6 mm; 130 × 130 pixel matrix; 84 interleaved axial slices aligned to the anterior-posterior commissure line] (Setsompop *et al.*, 2012). The total scan sequence corresponded to 628 whole-brain EPI volumes. A T1-weighted high-resolution anatomical image using a magnetization-prepared 2 rapid gradient echo sequence (MP2RAGE; Marques *et al.*, 2010) was acquired for each participant to assist with functional time-series co-registration [TR = 5000 ms; TE, 3.0 ms; inversion time, 700/2700 ms; pulse/flip angle, 4/5°; field of view, 24 cm; slice thickness (no gap), 0.73 mm; 330 × 330 pixel matrix; 84 sagittal slices aligned parallel to the midline]. To assist with head immobility, foam padding inserts were placed on either side of the participants’ head. Cardiac and respiratory recordings were sampled at 50 Hz using a pulse oximeter and respiratory belt. Information derived from these recordings was used for physiological noise correction (see further).

### Image pre-processing

Imaging data were pre-processed using Statistical Parametric Mapping (SPM) 12 (v7771, Wellcome Trust Centre for Neuroimaging, London, UK) within a MATLAB 2019b environment (The MathWorks Inc., Natick, MA). Motion artifacts were corrected by realigning each participant’s time series to the mean image, and all images were resampled using fourth-degree B-spline interpolation. Individualized motion regressors were created within SPM to account for movement. Four participants were excluded due to a mean total scan-to-scan displacement over 1.6 mm (i.e. the size of one voxel). Each participant’s anatomical images were co-registered to their respective mean functional image, segmented and normalized to the International Consortium of Brain Mapping template using the unified segmentation plus Diffeomorphic Anatomical Registration Through Exponentiated Lie Algebra (DARTEL) approach. Smoothing was applied with a 3.2 mm^3^ full-width-at-half-maximum Gaussian kernel to preserve spatial specificity.

Physiological noise was modeled at the first level using the PhysIO Toolbox (Kasper *et al.*, 2017). This toolbox applies noise correction to fMRI sequences using physiological recordings and has been found to improve blood-oxygen-level-dependent (BOLD) signal sensitivity and temporal signal-to-noise ratio at 7T (Reynaud *et al.*, 2017; see also Steward *et al.*, 2022). The Retrospective Image-based Correction function (Glover *et al.*, 2000) was applied to model the periodic effects of heartbeat and breathing on BOLD signals, using acquired cardiac/respiratory phase information. The respiratory response function (Birn *et al.*, 2008), convolved with respiration volume per time, was used to model low-frequency signal fluctuations, which arose from changes in breathing depth and rate. Heart rate variability was convolved with a pre-defined cardiac response function (Chang *et al.*, 2009) to account for BOLD variances due to heart rate–dependent changes in blood oxygenation. Individualized DARTEL tissue maps segmented from each participant’s respective anatomical scan were used to apply aCompCor, which modeled negative BOLD signals using six principal components derived from white matter and cerebrospinal fluid (Behzadi *et al.*, 2007).

### General linear modeling

Participants’ pre-processed time-series and nuisance regressors (i.e. physiological noise and motion fingerprint regressors) were included in the general linear modeling (GLM) analysis, with the onset times for each condition event specified and convolved with the SPM canonical hemodynamic response function. A 128 Hz high-pass filter was applied to account for low-frequency noise. Temporal autocorrelation was estimated using SPM’s FAST method, which has been shown to outperform AR(1) at short TRs and yield superior reliability (Olszowy *et al.*, 2019). First-level (single-subject) contrast images were then estimated to characterize brain responses during the cognitive restructuring relative to repeating statements mentally. Contrasts were estimated for cognitive restructuring generally (CHAL > REP) and to disentange the specific regions that exhibit greater activation during the restructuring of self-judgment beliefs relative to social-judgment beliefs and vice versa (i.e. contrasts of CHAL_SELF_ > CHAL_SOCIAL_ and CHAL_SOCIAL_ > CHAL_SELF_).

Contrast images for each participant were entered into second-level (group) random-effects GLMs (one-sample *t*-tests). For all analyses, significance thresholds corrected for multiple comparisons were calculated at the cluster level using threshold-free cluster enhancement (TFCE) using the TFCE toolbox in SPM (Gaser, 2019). In TFCE, voxel-wise values take into account the activation of neighboring voxels, which provides greater sensitivity to detecting low-amplitude, spatially distributed activation, as well as more focal signals (Smith and Nichols, 2009). This method utilizes an algorithm that enhances areas of the neural signal that have cluster-like support relative to background noise, aiming to better discriminate between spatially extended signal and noise. The algorithm determines the optimal threshold of signal intensity and cluster size, overcoming the need for arbitrary researcher-defined cluster-forming thresholds (Smith and Nichols, 2009). A conservative family-wise error (FWE)–corrected threshold (*P*_FWE_ < 0.05) was determined for each whole-brain analysis using 5000 permutations per test, in addition to a 10 voxel cluster-extent threshold (KE ≥ 10 voxels). These analyses were used both as primary tests of the hypotheses regarding the neural circuitry of cognitive restructuring and to identify specific regions that are more active during the restructuring of social- and self-judgment beliefs. All GLM results are presented on the MNI152 T1 0.5 mm template.

### Psychophysiological interaction analyses

A voxel of interest (VOI) was extracted from the peaks of the main clusters that showed significantly greater activation during the restructuring of social-judgment and of self-judgment beliefs, i.e. from the dorsal PCC/precuneus (*x* = 3, *y* = −58, *z* = 32) and the ventral PCC/retrosplenial cortex (*x* = −6, *y* = −56, *z* = 16), respectively. VOIs were extracted with a 4 mm sphere around the peak voxel. While the GLM analyses explored which regions showed greater activation during the above task contrasts, these subsequent psychophysiological interaction (PPI) analyses aimed to investigate how the dorsal and ventral PCC subregions interacted with the broader regions of the cognitive restructuring network. In this way, the PPI analyses were not biased by the differential involvement of the PCC subregions in self-belief *vs* social belief processing. To implement these analyses, we used an inclusive mask of the overall ‘CHAL > REP’ contrast to constrain the test of PCC functional connectivity to only those regions implicated in overall cognitive restructuring. Overall, the intent of the PPI analyses was to supplement the initial GLM results by providing a specific further test of task-dependent functional connectivity (Friston *et al.*, 1997; Ashburner *et al.*, 2020). Significance thresholds were once again calculated at the cluster level using TFCE in SPM (Gaser, 2019), *P*_FWE_ < 0.05, 5000 permutations per test, KE ≥ 10 voxels.

## Results

### Behavioral data

Mean endorsement of self-judgment *vs* social-judgment self-belief statements did not differ pre-task (SelfMean = 2.65, SocialMean = 2.53, mean difference = 0.12, *t* = 1.18, *P* = 0.24) or post-task (SelfMean = 2.04, SocialMean = 1.89, mean difference = 0.15, *t* = 1.50, *P* = 0.139). Self-belief statements that were challenged were significantly less endorsed post-scan compared to pre-scan for both self-judgment (mean difference = 0.60, *t* = 6.41, *P* < 0.001) and social-judgment (mean difference = 0.63, *t* = 8.25, *P* < 0.001) self-beliefs, suggesting that participants successfully engaged in Socratic questioning. While participants were slightly more likely to choose to challenge self-judgment (mean = 4.66 choices) relative to social-judgment (mean = 3.49 choices) statements (*t* = 4.76, *P* < 0.001), there was no difference in participants’ success in challenging beliefs, i.e. the mean pre-post changes in endorsement, between the two belief types (mean difference = 0.03, *t* = 0.30, *P* = 0.763). Further statistics for individual belief statements are presented in Supplementary Table S1.

### GLM

#### Challenging vs repeating negative self-beliefs

**Fig. 2.**
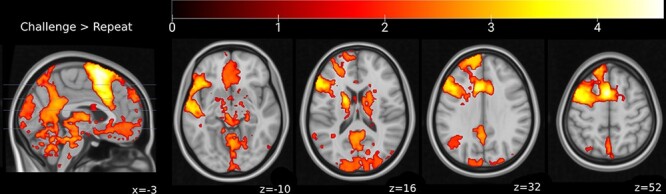
Brain regions that showed significant increases in activity during restructuring of negative self-beliefs relative to repeating. Figures reflect log-scaled FWE-corrected *P*-values for non-parametric TFCE values for the given contrast. Significance thresholds calculated at the cluster level using TFCE in SPM (Gaser, 2019), *P*_FWE_ < 0.05, 5000 permutations per test, KE ≥ 10 voxels. Results displayed on the MNI152 T1 0.5 mm template.

We first examined the neural circuitry involved in cognitive restructuring broadly, by investigating the brain regions that displayed greater activation during challenging relative to repeating of negative beliefs (CHAL > REP; Figure 2). As expected, challenging of negative self-beliefs was associated with activation in key cortical regions of the DMN, comprising the mPFC, specifically its ventromedial (vmPFC) and dorsomedial (dmPFC) aspects, the PCC extending to retrosplenial cortex and precuneus, the IPL (angular gyrus) and the anterior insula. Restructuring was also associated with greater activation in distributed frontoparietal cortical regions, including the dorsal anterior cingulate cortex (dACC) extending to the supplementary motor area (SMA) and superior frontal gyrus, together with the dorsolateral prefrontal cortex (dlPFC) and ventrolateral prefrontal cortex (vlPFC, including the inferior frontal gyrus). Subcortically, significantly greater activation was observed in the mediodorsal and pulvinar thalamus, as well as the cerebellum (Crus I and Lobule VI), and the putamen, extending into the caudate and pallidum. Further results are depicted in Supplementary Table S2.

### Challenging self- *vs* social-judgment negative self-beliefs

**Fig. 3.**
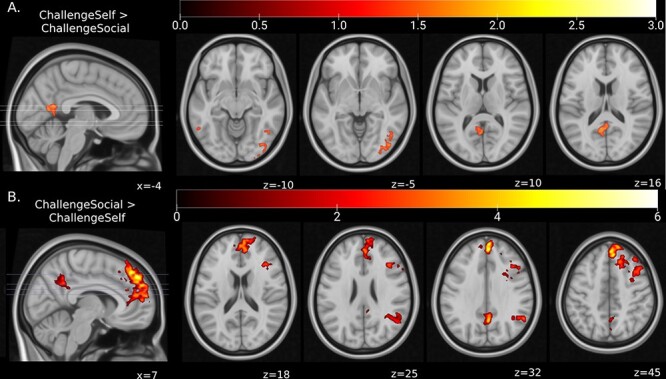
Brain regions that showed significant increases in activity during (**A**) restructuring of self-judgment *vs* social-judgment beliefs (CHAL_SELF_ > CHAL_SOCIAL_) and (**B**) restructuring of social-judgment *vs* self-judgment beliefs (CHAL_SOCIAL_ > CHAL_SELF_). Figures reflect log-scaled of FWE-corrected *P*-values for non-parametric TFCE values for the given contrast. Significance thresholds calculated at the cluster level using TFCE in SPM (Gaser, 2019), *P*_FWE_ < 0.05, 5000 permutations per test, KE ≥ 10 voxels. Results displayed on the MNI152 T1 0.5 mm template.

Challenging of self- relative to social-judgment beliefs (i.e. CHAL_SELF_ > CHAL_SOCIAL_) elicited greater activation in the ventral PCC/retrosplenial cortex and was also associated with activation in clusters spanning a broad range of occipitotemporal regions—comprising the inferior and middle occipital and temporal gyri, lingual gyrus, fusiform gyrus and cerebellum (Figure 3). Conversely, challenging of social-judgment statements (i.e. CHAL_SOCIAL_ > CHAL_SELF_) was associated with greater activation in the right dorsal PCC/precuneus, extending into the middle cingulate, the IPL (angular gyrus), vmPFC and vlPFC (including the inferior frontal gyrus). Challenging of social-judgment beliefs was also characterized by comparatively greater activation across distributed frontoparietal and temporal regions, including the SMA and pre-SMA, anterior insula, dACC, dlPFC, dmPFC and superior and middle temporal gyri. Further results are presented in Supplementary Table S3.

### Functional connectivity analyses

**Fig. 4.**
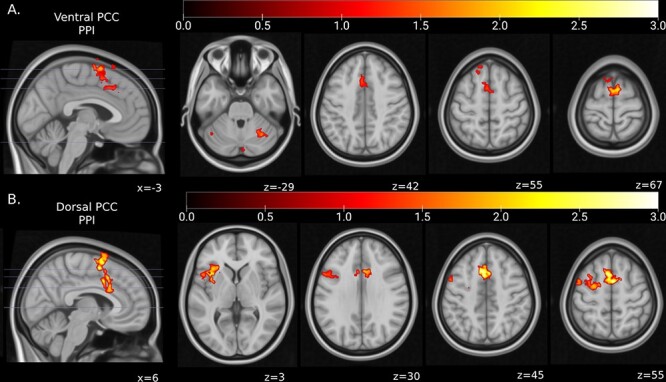
Brain regions that showed significant increases in functional connectivity with the (**A**) ventral PCC/retrosplenial cortex and (**B**) dorsal PCC/precuneus-based seed regions during cognitive restructuring. Figures reflect log-scaled FWE-corrected *P*-values for non-parametric TFCE values for the given contrast. Significance thresholds calculated within an inclusive mask of the CHAL > REP contrast, calculated at the cluster level using TFCE in SPM (Gaser, 2019), *P*_FWE_ < 0.05, 5000 permutations per test, KE ≥ 10 voxels. Results displayed on the MNI152 T1 0.5 mm template.

Functional connectivity analyses using PPI were conducted to examine how seed regions that showed greater activation during restructuring of social- or self-judgment negative beliefs act in concert with other brain regions in order to support cognitive restructuring more broadly (Figure 4). The seed regions in both the ventral PCC/retrosplenial cortex and dorsal PCC/precuneus, which were more active during the restructuring of self-judgment and social-judgment beliefs, respectively, had increased task-dependent functional connectivity with the bilateral SMA and pre-SMA and the left anterior middle cingulate cortex and medial superior frontal gyrus. The ventral PCC/retrosplenial cortex additionally showed greater connectivity with the right cerebellar Crus I and Lobule VI, while the dorsal PCC/precuneus seed exhibited increased functional connectivity with bilateral dorsolateral superior frontal gyrus and the ACC, as well as the left anterior insula, inferior frontal gyrus, lateral orbital gyrus, precentral gyrus and middle frontal gyrus. Full results are shown in Supplementary Table S4.

## Discussion

This study used ultra-high-field 7T fMRI to characterize the neural basis of cognitive restructuring of self- and social- judgment negative self-beliefs. Results supported our hypotheses that cognitive restructuring would be associated with activation in core regions of the DMN associated with self-referential cognition, along with frontoparietal control regions. We also observed that restructuring of self- *vs* social-judgment statements was primarily distinguished by differential involvement of the PCC. While core PCC involvement was involved in the challenging of both statement types as anticipated, contrary to expectations, we observed that there was greater activation in the ventral PCC/retrosplenial cortex during challenging of self-judgment statements and in the dorsal PCC/precuneus during challenging of social-judgment statements. Our connectivity analyses found evidence that both the ventral PCC/retrosplenial cortex and the dorsal PCC/precuneus display increased task-dependent functional connectivity with the SMA, pre-SMA and middle cingulate, while the dorsal PCC/precuneus also exhibited increased connectivity with regions involved in salience and attention, social cognition and mentalizing. This study is the first to highlight the differential engagement of the PCC during the restructuring of negative self-beliefs involving direct *vs* reflected self-appraisal.

The overall neural signature of cognitive restructuring that was observed in the present study aligns closely with previous literature investigating the restructuring of self-beliefs. It has been suggested that subregions of the DMN, chiefly the mPFC and PCC, act in concert with salience and valuation networks to support and direct attention toward mental representations of the self, while lateral and frontoparietal control regions support the modification of these self-representations (Goldin *et al.*, 2009; Dixon *et al.*, 2020). Previous work has highlighted that deficient engagement of frontoparietal regions during reappraisal of self-beliefs—and consequent hyper-engagement of the DMN—forms a neural signature of anxiety (Goldin *et al.*, 2009; Dixon *et al.*, 2020), suggesting that adaptive coordination of these regions is essential to the flexible manipulation of self-representations to maintain healthy self-concept. Activation was also identified in the regions of subcortex, notably the mediodorsal thalamus, whose broad excitatory pathways allow it to synchronize activity between large-scale DMN and prefrontal networks and sustain the engagement and synchrony of cortical function during task-focused states that give rise to complex mental self-representations (Greene *et al.*, 2020; Harrison *et al.*, 2022; Steward *et al.*, 2022). Collectively, these findings support a neural model of cognitive restructuring, whereby DMN regions work in concert with salience networks to maintain and direct attentional resources to mental self-representations that are manipulated by frontoparietal control regions, while these disparate networks are coordinated and dynamically modulated by the mediodorsal thalamus (Steward *et al.*, 2022).

Our findings further highlight the contrasting involvement of the PCC during the challenging of self- and social-judgment self-beliefs, consistent with previous evidence of functional and cytoarchitectural distinctions between the PCC’s ventral and dorsal subcomponents (Vogt and Laureys, 2005; Johnson *et al.*, 2006; Leech *et al.*, 2011; Leech and Sharp, 2014). Challenging self-judgment statements was associated with comparatively greater activation in the ventral PCC/retrosplenial cortex. The ventral PCC is known to play a critical role in supporting the coordination of mental representations during internally directed thought. In accordance with its dense anatomical connections with distributed networks throughout the brain (Leech and Sharp, 2014), the ventral PCC has been hypothesized to act as the central conduit through which temporoparietal cortical self-representations are gated into conscious awareness by prefrontal cortical networks, including the mPFC (Davey *et al.*, 2016). The dmPFC also exhibited greater activation during cognitive restructuring. However, our study further established that its activation was greater during challenging of social- relative to self-judgment beliefs, suggesting that it may play a more specialized role in the processing or manipulation of self-beliefs characterized by reflected self-appraisal. Although the specific mechanisms underlying this process are unclear, this could indicate that reflected self-appraisal necessitates the use of one’s own self-representations as a reference point from which others’ appraisals can be inferred (Lombardo *et al.*, 2010), requiring a more active or temporally extended involvement of the dmPFC (Tan *et al.*, 2022).

Findings from our connectivity analyses suggested that the ventral PCC had increased functional connectivity with the SMA and pre-SMA during cognitive restructuring. These regions may act to support metacognitive awareness during self-referential judgments, allowing individuals to consciously compare their own self-appraisals (coordinated by the ventral PCC) to the belief statements in question (Dixon *et al.*, 2020), facilitate the top-down modulation of activity in other networks and assist in the manipulation of self-representations during restructuring (Steward *et al.*, 2022). Additionally, greater connectivity was observed between the ventral PCC and Crus I and Lobule VI components of the cerebellum. Both Crus I and Lobule VI have been suggested to interact with core components of the salience network (including the dACC, dlPFC and insula) to integrate and filter relevant interoceptive and emotional information (Seeley *et al.*, 2007; Habas *et al.*, 2009), particularly during emotion regulation (Cattaneo *et al.*, 2021; Frazier *et al.*, 2022), while Crus I has also been implicated in information updating and abstract reasoning (Collette *et al.*, 2007). Interactions of these cerebellar subregions with the ventral PCC may similarly play a role in evaluating the valence and self-relevance of negative belief statements and updating cognitive representations of the self.

Conversely, we additionally identified that restructuring of social-judgment statements relative to self-judgment statements was supported by heightened activity in the dorsal PCC/precuneus, in conjunction with core nodes of the DMN and regions involved in mentalizing and social cognition. Within the framework of the neural model of cognitive restructuring described earlier, previous social neuroscience literature highlights a multifaceted role of the dorsal PCC in task-relevant domains. It has been elsewhere proposed that the dorsal PCC supports mentalizing about the actions and thoughts of others, including inference of others’ mental states (Spunt *et al.*, 2011; Bellucci *et al.*, 2020). However, these findings are partially at odds with previous research suggesting that perspective shifting in reflected self-appraisal might be supported by interactions between the IPL and ventral PCC (Delahoy *et al.*, 2022). It is possible that both the ventral PCC and dorsal PCC play a role in facilitating reflected self-appraisal more broadly, but the dorsal PCC is more specifically involved in facilitating the cognitive manipulation of social-judgment beliefs.

This parcellation of the PCC subcomponents converges with previous frameworks that have delineated the roles of smaller distributed functional networks within the DMN. Two distinct DMN subsystems supporting different forms of cognition that differentially engage the posteromedial cortex have been identified, noting specifically that a ‘Network A’ including the ventral PCC may play a role in self-referent episodic projection, including remembering of past and prospection of future events, while ‘Network B’, which more focally involves the dorsal PCC, may be preferentially recruited during tasks involving mentalizing and theory of mind (Braga *et al.*, 2019; DiNicola *et al.*, 2020).

Indeed, findings from our connectivity analyses shed additional light on the functional role of the dorsal PCC within the broader neural signature of cognitive restructuring. The dorsal PCC also exhibited increased connectivity with regions involved with salience and social cognition. It has been suggested that the dorsal PCC may facilitate information flow between the DMN, frontoparietal control networks and salience networks in accordance with cognitive demands (Andrews-Hanna *et al.*, 2010; Leech *et al.*, 2011; Andrews-Hanna *et al.*, 2014; Leech and Sharp, 2014). In the context of cognitive restructuring, the dorsal PCC may act in conjunction with components of the salience network (chiefly the anterior insula and ACC) to flexibly switch between internal and external environments and allocate attentional resources to self-representations that are coordinated by the PCC (Menon and Uddin, 2010; Leech *et al.*, 2011; Davey *et al.*, 2016). In turn, the dorsal PCC modulates activity in the SMA and pre-SMA, which supports the evaluation of self-traits in relation to negative belief statements and assists in updating self-representations with new information (Dixon *et al.*, 2020; Steward *et al.*, 2022). Notably, the dorsal PCC and anterior insula have also been posited to facilitate switching between the DMN and social cognition networks more broadly (Mars *et al.*, 2012; Molnar-Szakacs and Uddin, 2013; Hu *et al.*, 2016; Uddin *et al.*, 2017). During the challenging of social-judgment beliefs, the PCC’s and anterior insula’s place at the nexus between these networks may also allow them to support the modification of self-beliefs by integrating information inferred from one’s social environment into mental representations of the self.

Interestingly, it has previously been proposed that as task demand increases, activation within the dorsal PCC synchronizes more closely with the DMN and anti-correlates with cognitive control and salience networks (Leech *et al.*, 2011). This notably contrasts with the findings of the present study, where the dorsal PCC instead exhibited increased connectivity with central nodes within each of these disparate functional networks. This discrepancy can likely be attributed to the complex demands of the task, which requires not only reflecting on one’s self-evaluations but also flexibly calling upon specific counter-examples from one’s internal and external (i.e. social) environments and integrating them into one’s own cognitive self-representations. The integration of these elements, as outlined earlier, necessitates a complex interplay between a series of self, salience, social and control networks, evidenced by the dorsal PCC’s broad functional connectivity profile during restructuring.

Consequently, the complex neural dynamics involved in challenging self-relevant beliefs carry implications for the broader affective science literature examining the cognitive regulation of emotions. Emotion regulation paradigms typically task participants with cognitively reframing (i.e. reappraising) their initial negative response to visually evocative stimuli that are not inherently self-relevant, e.g. an image of a car crash (McRae *et al.*, 2010; Buhle *et al.*, 2014; Schubert *et al.*, 2020). Cognitive reappraisal in this sense may involve reinterpreting the meaning of the stimulus, such as picturing that the depicted scenario may improve over time (McRae *et al.*, 2010)—in contrast to the cognitive restructuring techniques employed in the present study, where participants flexibly drew upon past experiences and factual evidence to counter belief statements. These studies reliably find activation of frontoparietal regions, which are purported to modulate activity in the areas involved in emotional experience (such as the amygdala; McRae *et al.*, 2010; Buhle *et al.*, 2014; Picó-Pérez *et al.*, 2017); however, these studies rarely see co-activation of the DMN as we and others have reported during challenging of self-relevant stimuli (Dixon *et al.*, 2020; Steward *et al.*, 2022). We would suggest that in everyday life, cognitive emotion regulation strategies are chiefly applied in response to self-relevant stimuli (e.g. reframing the interpretation of criticism one has received). Thus, this and other recent studies using self-relevant stimuli may provide brain activation patterns that more realistically reflect the real-world application of emotion regulation strategies compared to traditional reappraisal studies using images. The use of self-relevant stimuli may therefore be an important consideration for affective scientists aiming to investigate the neural basis of emotion regulation.

As such, this paradigm also uniquely positions the present study to reflect the real-world application of cognitive restructuring techniques in a clinical practice context. This study is among the first to investigate the neural mechanisms underpinning the challenging of self-referential beliefs and to directly highlight the differential engagement of internetwork interactions supporting self- *vs* social-judgment beliefs. In line with real-world cognitive restructuring practices, participants in the present study were trained in Socratic questioning techniques such as active rebuttal, reinterpretation and perspective shifting. As such, some slight variation in the specific restructuring approach used when challenging belief statements was a natural consequence. It may be of interest in future research to examine how specific components of cognitive restructuring may differ in their neural mechanisms, in line with more recent research in the emotion regulation literature that has examined different approaches to cognitive reappraisal of non-self-relevant stimuli (e.g. distancing via adopting a neutral third-party perspective of a situation *vs* reinterpreting the meaning of the stimuli in a more positive manner; Picó-Pérez *et al.*, 2017). Alternatively, future research may supplement the findings of the current study by examining the cognitive and behavioral factors informing participants’ decisions to choose specific cognitive restructuring approaches or indeed whether to challenge or repeat specific statements.

## Conclusions

Overall, our findings demonstrate a complex interplay of DMN, salience and frontoparietal control networks that are coordinated by the subcortex to support the cognitive restructuring of negative self-beliefs. In particular, we highlight differential patterns of PCC engagement between the cognitive restructuring of self-judgment and social-judgment negative self-beliefs. We propose that during cognitive restructuring, the dorsal PCC in particular plays a role in supporting dynamic interactions between the DMN and frontoparietal/salience networks, thereby facilitating flexible switching between internal and external environments, allocation of attentional resources and modification of self-representations that are coordinated by the PCC. Further work may examine how aberrant activation or internetwork connectivity of these PCC subregions may inhibit cognitive restructuring success or underpin symptomatology in psychiatric disorders.

## Supplementary Material

nsad024_Supp

## Data Availability

The data underlying this article will be shared on reasonable request to the corresponding author.
