# Supplementary material for: Differential engagement of the posterior cingulate cortex during cognitive restructuring of negative self- and social beliefs
Source: Soc Cogn Affect Neurosci. 2023 May 2;18(1):nsad024. doi: 10.1093/scan/nsad024 (PMC10182822; doi:10.1093/scan/nsad024)
Supplement: nsad024_Supp [file nsad024_supp.zip › scan-22-227-File002.docx]

Supplementary Table S1. Descriptive statistics for individual self-belief statements

| # | Statement | Type | Mean endorsement pre (SD) | Mean endorsement post (SD) | Challenged *n* (%) | Repeated *n* (%) |
| --- | --- | --- | --- | --- | --- | --- |
| 1 | I sometimes feel incompetent in the things I do | Self | 4.06 (1.41) | 2.61 (1.51) | 54 (66.7) | 27 (33.3) |
| 2 | I often feel like I don’t measure up to others | Social | 3.35 (1.43) | 2.13 (1.14) | 23 (28.4) | 58 (71.6) |
| 3 | I feel insignificant from time to time | Self | 3.48 (1.51) | 2.70 (1.44) | 44 (54.3) | 37 (45.7) |
| 4 | I usually think other people are more competent than I am | Social | 3.59 (1.30) | 2.26 (1.16) | 34 (42.0) | 47 (58.0) |
| 5 | I often think I will fail even if I make an effort | Self | 2.31 (1.20) | 1.46 (0.71) | 56 (69.1) | 25 (30.9) |
| 6 | I occasionally believe that that nobody will ever be attracted to me | Social | 2.58 (1.52) | 1.82 (1.11) | 55 (67.9) | 26 (32.1) |
| 7 | I usually believe that I’ll be rejected if people discover my flaws | Social | 3.02 (1.55) | 2.00 (1.06) | 37 (45.7) | 44 (54.3) |
| 8 | I repeatedly think that I’m a failure | Self | 2.26 (1.20) | 1.92 (1.11) | 51 (63.0) | 30 (37.0) |
| 9 | From time to time, I think I am boring and uninteresting | Self | 3.04 (1.76) | 1.77 (0.96) | 31 (38.3) | 50 (61.7) |
| 10 | I sometimes believe I am not good enough to be loved | Social | 2.40 (1.45) | 1.60 (0.96) | 43 (53.1) | 38 (46.9) |
| 11 | I commonly think that people want to take advantage of me | Social | 2.75 (1.63) | 2.05 (1.20) | 21 (25.9) | 60 (74.1) |
| 12 | I usually believe that people want me to fail | Social | 1.86 (1.02) | 1.76 (0.98) | 42 (51.9) | 39 (48.1) |
| 13 | I often think I am incapable of changing my life | Self | 2.14 (1.37) | 1.57 (1.05) | 60 (74.1) | 21 (25.9) |
| 14 | At times, I believe that I can’t do anything right | Self | 2.93 (1.49) | 2.38 (1.26) | 37 (45.7) | 44 (54.3) |
| 15 | I occasionally think I have little value as a person | Self | 2.27 (1.23) | 1.91 (1.16) | 47 (58.0) | 34 (42.0) |
| 16 | I frequently believe that people will hurt me in order to get what they need | Social | 2.35 (1.49) | 1.60 (0.89) | 30 (37.0) | 51 (63.0) |

Supplementary Table S2. Brain regions with greater activation during challenging relative to repeating of statements (CHAL>REP)

| Cluster size (K_E_) 1.6mm^3^ voxels | TFCE | P_FWE_ | Peak MNI coordinates | | | Peak structure | Cluster structures | |  |
| --- | --- | --- | --- | --- | --- | --- | --- | --- | --- |
|  |  |  | x | y | z |  |  |  |  |
| **CHAL>REP** | | | | | | | | | |
| 96344 | 9252.46 | <.001 | -3 | 6 | 70 | Supplementary motor area (L) | | Supplementary motor area (R), superior frontal gyrus – dorsolateral (L) | |
|  | 9228.51 | <.001 | -6 | 13 | 69 | Supplementary motor area (L) | | Superior frontal gyrus – dorsolateral (L), supplementary motor area (R), pre-supplementary motor area (L, R) | |
|  | 9118.38 | <.001 | -5 | 10 | 61 | Supplementary motor area (L) | | Supplementary motor area (R), superior frontal gyrus – dorsolateral (L), pre-supplementary motor area (L, R) | |
|  | 9099.09 | <.001 | -10 | 14 | 62 | Supplementary motor area (L) | | Superior frontal gyrus – dorsolateral and medial (L), pre-supplementary motor area (L, R) | |
|  | 8970.44 | <.001 | -10 | 16 | 72 | Supplementary motor area (L) | | Superior frontal gyrus – dorsolateral (L), pre-supplementary motor area (L, R) | |
|  | 8924.85 | <.001 | -3 | 10 | 51 | Supplementary motor area (L) | | Supplementary motor area (R), superior frontal gyrus – dorsolateral (L), middle cingulate (L, R) | |
|  | 8690.33 | <.001 | -5 | 18 | 62 | Supplementary motor area (L) | | Supplementary motor area (R), superior frontal gyrus – dorsolateral and medial (L) | |
|  | 7718.72 | <.001 | -13 | 6 | 72 | Superior frontal gyrus – dorsolateral (L) | | Supplementary motor area (L), pre-supplementary motor area (L, R) | |
|  | 7210.88 | <.001 | -10 | 2 | 74 | Supplementary motor area (L) | | Superior frontal gyrus – dorsolateral (L), precentral gyrus (L) | |
|  | 6957.05 | <.001 | 0 | 16 | 45 | Supplementary motor area (L) | | Supplementary motor area (R), middle cingulate (L, R), superior frontal gyrus – medial (L, R) | |
|  | 6689.93 | <.001 | -6 | 0 | 66 | Supplementary motor area (L) | | Superior frontal gyrus – dorsolateral (L), Supplementary motor area (R), precentral gyrus (L) | |
|  | 6654.93 | <.001 | 8 | 10 | 70 | Supplementary motor area (R) | | Supplementary motor area (L), superior frontal gyrus – dorsolateral (R) | |
|  | 6637.27 | <.001 | 11 | 5 | 72 | Supplementary motor area (R) | | superior frontal gyrus – dorsolateral (R) | |
|  | 6423.83 | <.001 | -3 | -2 | 75 | Supplementary motor area (L) | | Supplementary motor area (R), superior frontal gyrus – dorsolateral (L), paracentral lobule (L) | |
|  | 6406.88 | <.001 | 0 | 18 | 37 | Middle cingulate (L) | | Middle cingulate (R), superior frontal gyrus – medial (L, R), supplementary motor area (L, R), anterior cingulate (L, R) | |
|  | 6399.26 | <.001 | -6 | 18 | 32 | Middle cingulate (L) | | Anterior cingulate (L), superior frontal gyrus – dorsolateral and medial (L), middle cingulate (R), anterior cingulate (R) | |
| 517 | 857.63 | .009 | 50 | 10 | -24 | Middle temporal gyrus (R) | | Superior temporal gyrus (R), posterior orbital gyrus (R) | |
|  | 855.63 | .009 | 48 | 18 | -29 | Superior temporal gyrus (R) | | Middle temporal gyrus (R) | |
|  | 846.12 | .009 | 46 | 13 | -27 | Superior temporal gyrus (R) | | Middle temporal gyrus (R), inferior temporal gyrus (R), posterior orbital gyrus (R) | |
|  | 769.42 | .010 | 50 | 19 | -22 | Superior temporal gyrus (R) | | Middle temporal gyrus (R), posterior orbital gyrus (R), lateral orbital gyrus (R), ventrolateral prefrontal cortex (inferior frontal gyrus; R) | |
|  | 594.06 | .015 | 54 | -2 | -21 | Middle temporal gyrus (R) | | Superior temporal gyrus (R), inferior temporal gyrus (R) | |
|  | 576.58 | .016 | 58 | 11 | -16 | Superior temporal gyrus (R) | | Middle temporal gyrus (R), posterior orbital gyrus (R), insula (R), ventrolateral prefrontal cortex (inferior frontal gyrus; R) | |
|  | 561.04 | .017 | 56 | 5 | -14 | Superior temporal gyrus (R) | | Middle temporal gyrus (R), insula (R) | |
|  | 477.10 | .025 | 45 | 11 | -32 | Middle temporal gyrus (R) | | Superior temporal gyrus (R), inferior temporal gyrus (R) | |
| 261 | 856.96 | .009 | 43 | -37 | 2 | Superior temporal gyrus (R) | | Middle temporal gyrus (R), hippocampus (R), Heschl’s gyrus (R) | |
| 17 | 473.16 | .026 | -8 | 27 | -22 | Gyrus rectus (L) | | Medial orbital gyrus (L), superior frontal gyrus (L), olfactory gyrus (L), posterior orbital gyrus (L) | |
| 17 | 461.10 | .029 | -56 | -45 | 21 | Superior temporal gyrus (L) | | Supramarginal gyrus (L), middle temporal gyrus (L), angular gyrus (L) | |

Supplementary Table S3. Brain regions with greater activation during challenging of social-judgment statements relative to self-judgment statements (CHAL_SOCIAL_>CHAL_SELF_) and vice-versa (CHAL_SELF_>CHAL_SOCIAL_)

| Cluster size (K_E_) 1.6mm^3^ voxels | TFCE | P_FWE_ | Peak MNI coordinates | | | Peak structure | Cluster structures | |  |
| --- | --- | --- | --- | --- | --- | --- | --- | --- | --- |
|  |  |  | x | y | z |  |  |  |  |
| **CHAL_SELF_>CHAL_SOCIAL_** | | | | | | | | | |
| 423 | 829.76 | .003 | -6 | -56 | 16 | Precuneus (L) | | Calcarine (L, R), posterior cingulate (L), cuneus (L), precuneus (R) | |
|  | 782.88 | .006 | 0 | -58 | 11 | Precuneus (L) | | Calcarine (L, R), Lobule IV/V of vermis, precuneus (R), lingual gyrus (L, R), cuneus (R) | |
|  | 756.32 | .009 | 2 | -51 | 16 | Precuneus (R) | | Precuneus (L), posterior cingulate (L, R), calcarine (L, R) | |
| 888 | 780.68 | .006 | 42 | -78 | -3 | Inferior occipital gyrus (R) | | Middle occipital gyrus (R), inferior temporal gyrus (R), middle temporal gyrus (R), fusiform gyrus (R) | |
|  | 777.55 | .007 | 30 | -88 | -3 | Inferior occipital gyrus (R) | | Middle occipital gyrus (R), lingual gyrus (R), fusiform gyrus (R), calcarine (R), superior occipital gyrus (R) | |
|  | 775.51 | .007 | 40 | -86 | -2 | Inferior occipital gyrus (R) | | Middle occipital gyrus (R), fusiform gyrus (R) | |
| 32 | 706.26 | .018 | 11 | -46 | 2 | Lingual gyrus (R) | | Precuneus (R), Lobule IV/V of vermis, Crus IV/V of cerebellum, posterior cingulate (R), calcarine (R), Lobule III of vermis, hippocampus (R), parahippocampal gyrus (R) | |
| 29 | 662.21 | .032 | -54 | -54 | -10 | Inferior temporal gyrus (L) | | Middle temporal gyrus (L), inferior occipital gyrus (L) | |
| **CHAL_SOCIAL_>CHAL_SELF_** | | | | | | | | | |
| 4691 | 1412.88 | <.001 | 5 | 50 | 42 | Dorsomedial prefrontal cortex (superior frontal gyrus – medial; R) | Superior frontal gyrus – medial (L) and dorsolateral (R), pre-supplementary motor area (R), supplementary motor area (R) | |  |
|  | 1297.94 | <.001 | 3 | 48 | 32 | Dorsomedial prefrontal cortex (superior frontal gyrus – medial; R) | Superior frontal gyrus – medial (R), anterior cingulate cortex (R), middle cingulate (R),, ventrolateral prefrontal cortex (inferior frontal gyrus; R), anterior insula (R) | |  |
|  | 1292.41 | <.001 | 6 | 42 | 48 | Dorsomedial prefrontal cortex (superior frontal gyrus – medial; R) | Superior frontal gyrus – medial (L), superior frontal gyrus – dorsolateral ® | |  |
| 478 | 1156.32 | <.001 | 3 | -58 | 32 | Posterior cingulate (L) | Precuneus (L, R), posterior cingulate (R), middle cingulate (L, R) | |  |
|  | 1034.72 | <.001 | 2 | -56 | 42 | Precuneus (R) | Precuneus (L), middle cingulate (L, R), posterior cingulate (L) | |  |
|  | 688.74 | .024 | 8 | -43 | 27 | Posterior cingulate (R) | Middle cingulate (L, R), posterior cingulate (L), precuneus (R) | |  |
| 444 | 986.53 | .001 | 56 | -54 | 30 | Inferior parietal lobe (angular gyrus; R) | Supramarginal gyrus (R), inferior parietal gyrus (R), superior temporal gyrus (R) | |  |
|  | 869.54 | .003 | 48 | -54 | 32 | Inferior parietal lobe (angular gyrus; R) | Inferior parietal gyrus (R), supramarginal gyrus (R) | |  |
|  | 819.36 | .005 | 50 | -59 | 24 | Inferior parietal lobe (angular gyrus; R) | Middle temporal gyrus (R), middle occipital gyrus (R), superior temporal gyrus (R) | |  |
| 44 | 700.28 | .021 | 34 | 3 | 46 | Dorsolateral prefrontal cortex (middle frontal gyrus; R) | Precentral gyrus (R), superior frontal gyrus – dorsolateral (R) | |  |

Supplementary Table S4. Brain regions with increased functional connectivity with ventral PCC/retrosplenial cortex and dorsal PCC/precuneus voxels of interest (VOIs) during cognitive restructuring

| Cluster size  (K_E_) 1.6mm^3^ voxels | TFCE | P_FWE_ | Peak MNI coordinates | | | | | Peak structure | | Cluster structures |
| --- | --- | --- | --- | --- | --- | --- | --- | --- | --- | --- |
|  |  |  | x | y | | z | |  |  |  |
| **Ventral PCC VOI** | | | | | | | | | | |
| 501 | 541.35 | .007 | 6 | | 5 | | 67 | | Supplementary motor area (R) | Supplementary motor area (L), pre-supplementary motor area (R) |
|  | 495.94 | .011 | -3 | | 6 | | 67 | | Supplementary motor area (L) | Supplementary motor area (R), pre-supplementary motor area (R) |
|  | 438.86 | .023 | 5 | | -2 | | 77 | | Supplementary motor area (R) | Supplementary motor area (L), pre-supplementary motor area (R) |
| 67 | 437.79 | .021 | 30 | | -61 | | -29 | | Cerebellum Lobule VI (R) | Cerebellum Crus I (R), fusiform gyrus (R) |
| 47 | 420.10 | .026 | 8 | | -83 | | -21 | | Cerebellum Crus I (R) | Cerebellum Lobule VI (R), Vermis Lobule VII, lingual gyrus (R), Vermis Lobule VI, Cerebellum Crus II (L), Cerebellum Crus I (L) |
| 20 | 387.92 | .037 | -3 | | 16 | | 43 | | Supplementary motor area (L) | Middle cingulate (L), medial superior frontal gyrus (L, R), middle cingulate (R), superior frontal gyrus (L), supplementary motor area (R) |
| **Dorsal PCC VOI** | | | | | | | | | | |
| 3136 | 745.92 | .001 | -5 | 14 | | 53 | | Supplementary motor area (L) | | Superior frontal gyrus – medial and dorsolateral (L), supplementary motor area (R), pre-supplementary motor area (L, R), middle cingulate (L), anterior cingulate cortex (L) |
|  | 726.26 | .001 | 3 | 3 | | 56 | | Supplementary motor area (R) | | Supplementary motor area (L), pre-supplementary motor area (L, R), middle cingulate (L), anterior cingulate cortex (R) |
|  | 716.63 | .001 | -5 | -3 | | 59 | | Supplementary motor area (L) | | Supplementary motor area (R), pre-supplementary motor area (L, R), middle cingulate (L), anterior cingulate cortex (L) |
| 674 | 597.99 | .005 | -38 | 16 | | 2 | | Anterior insula (L) | | Inferior frontal gyrus – opercular, triangular, and pars orbitalis (L) |
|  | 537.73 | .009 | -45 | 11 | | 10 | | Inferior frontal gyrus – opercular (L) | | Anterior insula (L), inferior frontal gyrus – triangular (L), rolandic operculum (L), precentral gyrus (L) |
|  | 529.17 | .010 | -53 | 19 | | -5 | | Inferior frontal gyrus – triangular (L) | | Inferior frontal gyrus – opercular (L), superior temporal gyrus (L), insula (L), rolandic operculum (L) |
| 77 | 405.88 | .035 | -53 | 13 | | 30 | | Inferior frontal gyrus – triangular (L) | | Precentral gyrus (L), inferior frontal gyrus – opercular and triangular (L), dorsolateral prefrontal cortex (middle frontal gyrus; L) |
|  | 405.85 | .035 | -58 | 13 | | 19 | | Inferior frontal gyrus – opercular (L) | | Inferior frontal gyrus – triangular (L), precentral gyrus (L), postcentral gyrus (L) |
|  | 396.42 | .038 | -45 | 11 | | 29 | | Precentral gyrus (L) | | Inferior frontal gyrus – opercular (L), precentral gyrus (L), inferior frontal gyrus – triangular (L), dorsolateral prefrontal cortex (middle frontal gyrus; L) |
| 14 | 402.47 | .036 | -42 | -61 | | -29 | | Cerebellum Crus I (L) | | Cerebellum Lobule VI (L), fusiform gyrus (L), inferior temporal gyrus (L) |
| 14 | 385.20 | .043 | -50 | 11 | | 24 | | Inferior frontal gyrus – opercular (L) | | Inferior frontal gyrus – triangular (L), precentral gyrus (L) |
